# Mass irradiation of adult Aedes mosquitoes using a coolable 3D printed canister

**DOI:** 10.1038/s41598-024-55036-2

**Published:** 2024-02-22

**Authors:** F. Balestrino, N. S. Bimbilé Somda, M. Samuel, S. Meletiou, O. Bueno, T. Wallner, H. Yamada, W. Mamai, M. J. B. Vreysen, J. Bouyer

**Affiliations:** 1 Insect Pest Control Laboratory, Joint FAO/IAEA Centre of Nuclear Techniques in Food and Agriculture, IAEA, 1400 Vienna, Austria; 2 National Institute for Communicable Diseases, Centre for Emerging Zoonotic and Parasitic Diseases, Johannesburg, 2131 South Africa; 3 Unité de Formation et de Recherche en Science et Technologie (UFR/ST), Université Norbert ZONGO (UNZ), BP 376, Koudougou, Burkina Faso; 4 Department of Chemical Engineering, Cyprus University of Technology, 3020 Limassol, Cyprus; 5 UMR ASTRE, CIRAD, 34398 Montpellier, France

**Keywords:** Entomology, Infectious diseases

## Abstract

In the last decade, the use of the sterile insect technique (SIT) to suppress mosquito vectors have rapidly expanded in many countries facing the complexities of scaling up production and procedures to sustain large-scale operational programs. While many solutions have been proposed to improve mass production, sex separation and field release procedures, relatively little attention has been devoted to effective mass sterilization of mosquitoes. Since irradiation of pupae *en masse* has proven difficult to standardise with several variables affecting dose response uniformity, the manipulation of adult mosquitoes appears to be the most promising method to achieve effective and reliable sterilization of large quantities of mosquitoes. A 3D-printed phase change material based coolable canister was developed which can compact, immobilize and hold around 100,000 adult mosquitoes during mass radio sterilization procedures. The mass irradiation and compaction treatments affected the survival and the flight ability of *Aedes albopictus* and *Aedes aegypti* adult males but the use of the proposed irradiation canister under chilled conditions (6.7–11.3 °C) significantly improved their quality and performance. The use of this cooled canister will facilitate adult mass irradiation procedures in self-contained irradiators in operational mosquito SIT programmes.

## Introduction

Mosquito-borne diseases are currently among the most common causes of illness and death worldwide. Invasive Aedes mosquitoes are constantly expanding their distribution, contributing significantly to increase the global mortality and morbidity of infectious diseases such dengue, chikungunya, Zika and yellow fever^1^. The current vector control methods are unable to prevent epidemics and new sustainable effective tools are urgently needed to suppress mosquito vectors^2^. The sterile insect technique (SIT) is an environmentally friendly insect pest control method, which has been successfully applied to prevent, suppress and eradicate several major insect pest populations^3^. The SIT relies on the repeated inundative release of conspecific and competitive radiation sterilised males to progressively reduce the fertility in the wild population. In recent years, both the SIT and related genetic control methods have demonstrated their ability to effectively impact mosquito population density and dynamics, making them a potential sustainable tool that can be included into area-wide integrated vector management (AW-IVM) programs^4^. In many countries, the feasibility projects to evaluate the efficacy of the SIT against mosquito vectors have progressively moved from baseline data collection to small-scale field trials with experimental releases of sterile males, generally provided by laboratory-scale equipment and procedures^5^. However, in recent years many of these SIT projects have rapidly expanded and scaled up their production capacity by simply enlarging the usual laboratory and sterilization equipment and procedures. Among the significant challenges to progress towards larger-scale operational levels, there is a need to develop procedures and equipment that allow the consistent and sustainable mass-rearing, radiation sterilization and release of a larger number of competitive sterile male mosquitoes. While many technical guidelines and operating procedures have been formulated to improve the production, the sex separation and the release of large quantities of male mosquitoes in the field^6^, relatively little attention has been devoted to effectively manage the sterilization of large volumes of mosquitoes. Ionizing radiation is the most commonly used method to induce sterility in area-wide integrated pest management (AW-IPM) programmes with an SIT component^7^. Since its first application, radiation sterilization has proven to be a practical and safe method to induce sterility in mosquitoes^8^ mainly targeting the pupal stage submerged in water. However, upscaling pupal irradiation procedures presents challenges, as it involves handling large quantities of pupae of mixed ages in a short timeframe before emergence, often at very high densities. Conditions to irradiate pupae *en masse* have proven difficult to standardise due to the rapid depletion of dissolved oxygen in water by the pupal cuticular respiration, which increases during forced submersions^9^. Consequently, the irradiation time of pupae in these hypoxic, radioprotective conditions is prolonged leading to unpredictable sterilizing effects and decreased quality^10^. Although the management of large quantities of pupae may seem easier than for adults, effective sterilization procedures require tedious synchronization of the larval development to pupal stage, stable ambient conditions, and strict scheduling to efficiently manage production, sterilization and release procedures^11^. In contrast, the sterilization of adult mosquitoes requires the development of accurate and dedicated procedures but yields more consistent results and minimizes somatic damage whilst maximizing the effects on the germ cells^11,12^. To achieve reliable sterilization of large quantities of adult mosquitoes, it is therefore imperative to design and validate dedicated tools to compact and immobilize the fragile adults at high densities during the sterilization process, whilst minimizing the negative effects on the final quality of the sterile males produced. Chilling seems to be the most safe and efficient method for *Aedes* adult immobilization^13^. A temperature between 7 and 12 °C for a duration of up to 24 h has been reported to not negatively impact the survival and flight capabilities of *Aedes* adults^14–17^, while temperatures below 6 °C can lead to a strong reduction in the quality of the treated males^16,18^. Compaction of cold anaesthetised adult *Aedes* mosquitoes may also lead to a reduction in the overall insect quality. However, several studies have reported marginal effects on survival and flight ability when compacted at densities of 100–240 adult/cm^3^^11,14,16,19^. Based on available literature and evidence obtained through large-scale adult handling, packaging, transportation and release trials^20,21^, we developed a mass irradiation canister with a maximum depth of 4 cm allowing a compaction density of about 120 adult mosquito/cm^3^ while maintaining a temperature between 8 and 12 °C.

A 3D-printed canister, with cooling based on phase change material (PCM) was developed and tested at the Insect Pest Control Laboratory (IPCL) of the Joint FAO/IAEA Centre of Nuclear Techniques in Food and Agriculture to allow the bulk sterilization of adult *Aedes* mosquitoes for larger-scale SIT operational programmes. In this paper, we report the technical performance of the canister and the effects of thermal shock, compaction and cooling treatment on the quality and sterility of the adult males maintained inside the coolable canister during irradiation.

## Results

### Cooled irradiation canister

#### Effect of cold ring on temperature and relative humidity

**Figure 1:**
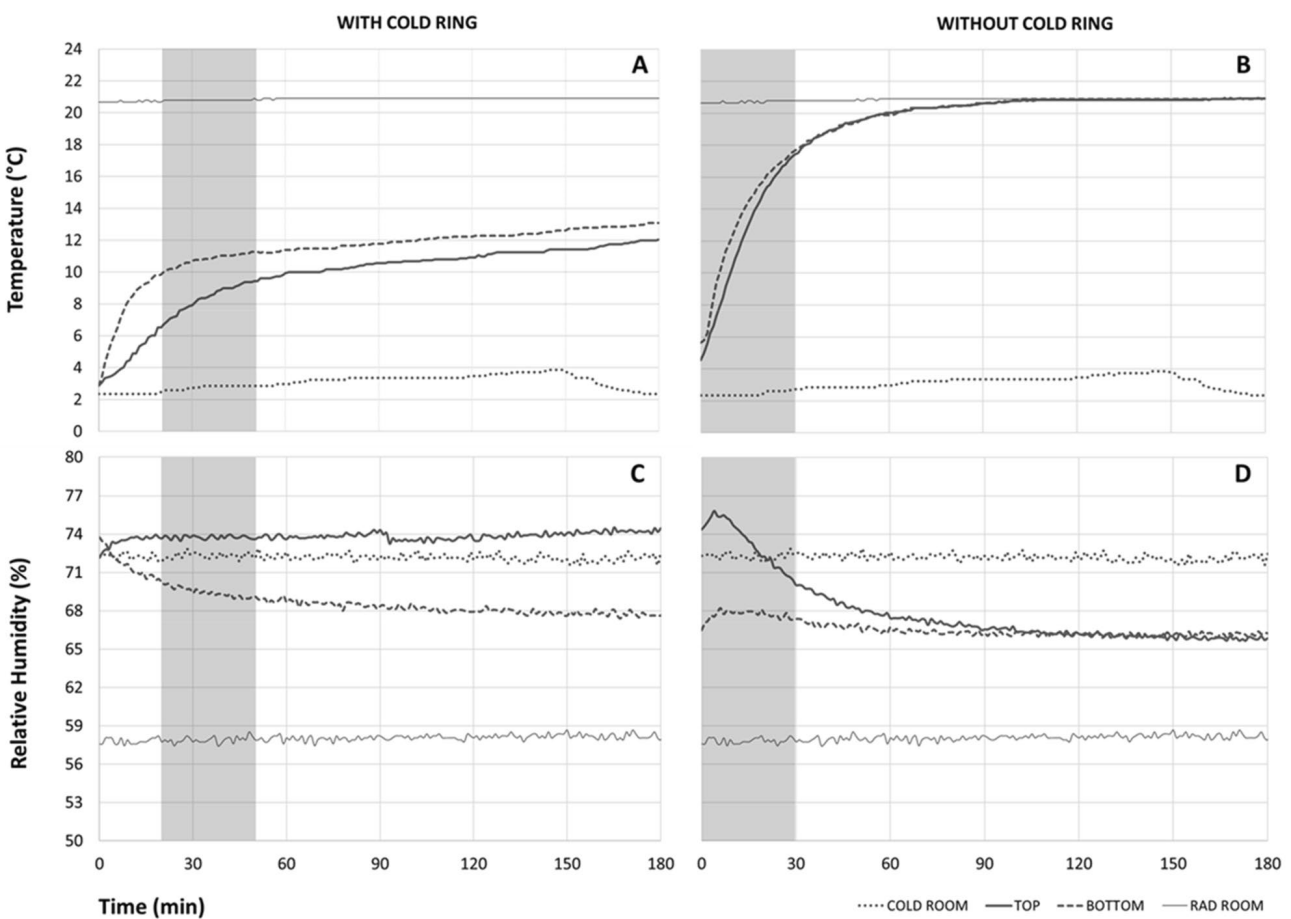
Trends of temperature (**A** and **B**) and relative humidity (**C** and **D**) in the lower (bottom) and upper (top) compartments of the canister with and without the use of the cold ring. The temperature and relative humidity parameters recorded in the radiation (rad room) and cold rooms (cold room) are also reported. The grey areas indicate the environmental conditions selected to test the cold ring during the 30-min trials using cold anesthetised mosquitoes.

A 3D plastic circular canister capable to compact and hold approximately 100,000 cold anesthetized adult mosquitoes during mass radio sterilization procedures was produced and tested. The canister consists of two overlapping compartments and can be inserted inside an external cold ring ensuring sustained chilling throughout the entire canister (for more details see Materials and Methods). The temperature and humidity in the mass irradiation canister filled with dead chilled mosquitoes and positioned inside a Gammacell irradiator (GC 220 Nordion Ltd., Kanata, Ontario, Canada) were recorded with and without the use of the cold ring. The presence of the cold ring (F_1,122_ = 36.2; *p* < 0.001) and the position of the mosquitoes in the different compartments of the canister (F_1,122_ = 9.7; *p* < 0.01) affected the temperature during the 30 min of the trial. When the canister filled with dead chilled mosquitoes was coupled with the cold ring, the temperature ranged between 2.0 and 13.5 °C in the first 3 h, gradually reaching room temperature after about 11 h (Fig. 1A). Temperatures below 6°C were observed in the canister compartments in the first 20 min after chilling suggesting the allowance of a brief warmup for the cold ring at room temperature before its use. After this initial time, different (F_1,60_ = 204.8; *p* < 0.001) thermal trends in the lower (bottom: T_MEAN_ = 10.8 °C; T_MIN—MAX_ = 9.8–11.3 °C) and upper (top: T_MEAN_ = 8.3 °C; T_MIN—MAX_ = 6.7–9.4 °C) sections of the canister were observed during 30 min after cold ring placement.

Without the use of the cold ring, the canister filled with dead chilled mosquitoes reached the external environmental parameters after only 1 h but maintained an effective chilling temperature between 6 and 12 °C^21^ in the first 10 min after the mosquito cold knockdown treatment (Fig. 1B). During the first 30 min of observation, a similar (F_1,60_ = 1.56; *p* = 0.216) thermal trend in the lower (bottom: T_MEAN_ = 14.1 °C; T_MIN—MAX_ = 6.2–17.9 °C) and upper (top; T_MEAN_ = 13.0 °C; T_MIN—MAX_ = 5.9–17.8 °C) compartments of the canister were observed.

The presence of the cold ring (F_1,122_ = 5.53; *p* < 0.05) and the position of the mosquitoes in the different compartments of the canister (F_1,122_ = 584.4; *p* < 0.001) affected the relative humidity during the 30-min trials. In this period the overall mean (± SD) relative humidity measured inside the canister filled with dead chilled mosquitoes with the use of the cold ring (71.6 ± 2.2%; Fig. 1C) was higher than the values observed in absence of the cold ring (70.5 ± 3.1%; Fig. 1D). Humidity was significantly higher in the upper sections of the canister in comparison with the lower compartments with (top: 73.8 ± 0.1% and bottom: 69.5.0 ± 0.4%; F_1,60_ = 3696.4; *p* < 0.001; Fig. 1C) and without (top: 73.3 ± 1.8% and bottom: 67.7 ± 0.3%; F _1,60_ = 288.2; p < 0.001; Fig. 1D) the use of the cold ring.

#### Effect of the cold ring on dose rate attenuation

Dose distribution, attenuation and uniformity obtained inside the mass irradiation canister with and without the use of the cold ring was investigated. The mean (± 95% CI) dose rate measured inside the canister with the use of the cold ring (43.88 ± 1.17 Gy/min) was significantly attenuated (F_1,18_ = 7.016; *p* < 0.05) in comparison with the dose rate observed using only the plastic canister (45.99 ± 1.37 Gy/min). However, an overall similar (F_1,18_ = 0.37; *p* = 0.551) mean (± 95% CI) dose rate was measured in the upper (44.81 ± 1.47 Gy/min) and lower positions (45.05 ± 1.52 Gy/min) of the compartments. The overall mean (± SD) attenuation measured in the canister with the use of the cold ring is equal to 4.79 ± 1.17%. The central portions of the upper and lower compartment show a lower dose rate compared to the respective peripheral areas irrespective of the use of the cold ring and remained consistent with the typical dose distribution of the GC220 gamma irradiator^22^. The dose uniformity is generally characterised by the parameter DUR (dose uniformity ratio) calculated as the ratio of the maximum dose to the minimum dose within the canister^7^. The DUR parameters measured in the canister with and without the use of the cold ring area were 1.11 and 1.13, respectively.

### Knockdown, compaction, cooling and irradiation treatments

#### Effect of treatments on male survival

**Figure 2:**
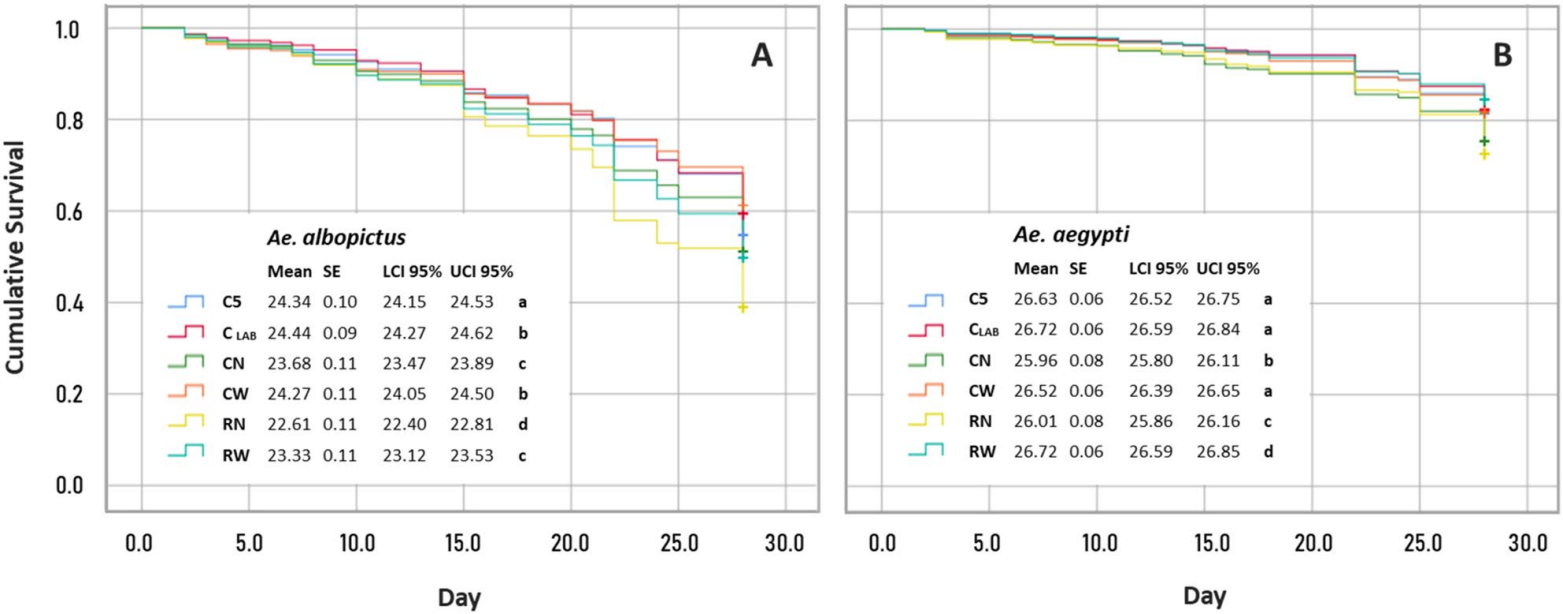
Survival curves of *Ae. albopictus* (**A**) and *Ae. aegypti* (**B**) males subjected to different treatments: C_LAB_, C_5_, C_W_, C_N_, R_W_ and R_N_. C_LAB_ untreated adult males, C_5_ cold knockdown adult males, C_W_ cold knockdown, compacted and cooled adult males; C_N_ cold knockdown and compacted adult males; R_W_ cold knockdown, compacted, cooled and irradiated adult males; R_N_ cold knockdown, compacted and irradiated adult males. Tables report the estimated mean survival time (in days) together with standard errors and 95% confidence intervals for the mean in each treatment groups. Different letters represent statistically significant differences of survival distributions at *p* < 0.05 level (Mantel-Cox log-rank test) among the different treatments.

Cold immobilised (knockdown treatment) Aedes adult males were compacted inside the plastic mass irradiation canister and subjected to additional cooling (cold ring) and/or irradiation treatments for 30 min. Results indicated that the irradiation treatment significantly increased the mortality rate of the adult *Ae. albopictus* males (coef = 0.353, *p* < 0.001; Table 1). An irradiated *Ae. albopictus* adult male had a 1.42-fold increase in hazard to death when subjected to irradiation (odds ratio or exp(coef) = 1.42; Table 1). The compaction and the cold knockdown treatments experienced by the adult mosquitoes during the process also negatively impacted *Ae. albopictus* adult survival but to a lesser extent than irradiation (exp(coef) = 1.16 and 1.07 respectively; Table 1). However, the use of the cold ring resulted in a significant improvement in the survival rate of the irradiated *Ae. albopictus* males reducing the hazard of the chilled adult by 1.34-fold (coef = − 0.295; exp(coef) = 0.74; Table 1). The mean (± SE) estimated survival for the compacted adult *Ae. albopictus* males under cooled conditions (C_W_ = 24.27 ± 0.11 day; Fig. 2A) was similar (χ^2^ = 2.26, *df* = 1, *p* = 0.132) to the survival observed for the untreated control (C_LAB_ = 24.44 ± 0.09 da; Fig. 2A) and the use of the cold ring (C_W_) significantly increases (χ^2^ = 74.26, *df* = 1, *p* < 0.001) the survival of the males under compaction conditions (C_N_ = 23.68 ± 0.11 day; Fig. 2A). The estimated survival time for the adult *Ae. albopictus* males irradiated in chilled conditions (R_W_ = 23.33 ± 0.11 day; Fig. 2A) was significantly lower (χ^2^ = 97.23, *df* = 1, *p* < 0.001) as compared with the untreated control (C_LAB_, Fig. 2A) but significantly higher (χ^2^ = 97.54, *df* = 1, *p* < 0.001) than the survival of the compacted males irradiated without the cold ring (R_N_ = 22.61 ± 0.11 day; Fig. 2A).

**Table 1:** Fixed coefficients of the Cox mixed-effects model for the effect of knockdown, compaction, cooling and irradiation on *Aedes albopictus* and *Aedes aegypti* male survival (***p < 0.001, **p < 0.01, *p < 0.05).

Species	Fixed coefficients	Coef	Exp(coef)	SE (coef)	z value	*p*
*Ae. albopictus*	radiation	0.353	1.423	0.022	16.26	0.000***
	cooling	− 0.295	0.744	0.022	− 13.71	0.000***
	compaction	0.149	1.161	0.029	5.19	0.000***
	knockdown	0.066	1.068	0.030	2.16	0.031*
*Ae. aegypti*	radiation	0.085	1.089	0.041	2.07	0.039*
	cooling	− 0.368	0.692	0.045	− 8.20	0.000***
	compaction	0.377	1.458	0.038	9.80	0.000***
	rad:cooling	− 0.205	0.814	0.066	− 3.13	0.002**

The irradiation treatment of *Ae. aegypti* significantly increased the hazard to death of the adult males (exp(coef) = 1.09; Table 1) but to a lesser extent than the compaction treatment (exp(coef) = 1.46; Table 1). Unlike what was observed in *Ae. albopictus*, the cold knockdown treatment of the adult mosquitoes did not significantly affect the survival of the adult *Ae. aegypti* males and was therefore not included in the final model. As observed in *Ae. albopictus*, the cold ring significantly reduced the hazard to death of the chilled adult males by 1.44-fold (exp(coef) = 0.69; Table 1), especially for those subjected to the radiation treatment (exp(coef) = 0.81; Table 1). Similarly to *Ae. albopictus*, the mean estimated survival time for adult *Ae. aegypti* males compacted under cooled conditions (C_W_ = 26.52 ± 0.06 day; Fig. 2B) was similar (χ^2^ = 0.993, *df* = 1, *p* = 0.319) as compared with the untreated control (C_LAB_ = 26.72 ± 0.06 day; Fig. 2B) and significantly higher (χ^2^ = 54.49, *df* = 1, *p* < 0.001) than the survival of the males compacted without the use of the cold ring (C_N_ = 25.96 ± 0.08 day; Fig. 2B). The estimated survival time for the adult *Ae. aegypti* males irradiated under cooled conditions (R_W_ = 26.72 ± 0.06 day; Fig. 2B) was significantly lower (χ^2^ = 6.16, *df* = 1, *p* < 0.05) as compared with the untreated control (C_LAB_, Fig. 2B) but significantly higher (χ^2^ = 174.47, *df* = 1, *p* < 0.001) than the mean survival time of the compacted males irradiated without the cold ring (R_N_ = 26.01 ± 0.08 day; Fig. 2B).

#### Effect of treatments on male flight ability

**Figure 3:**
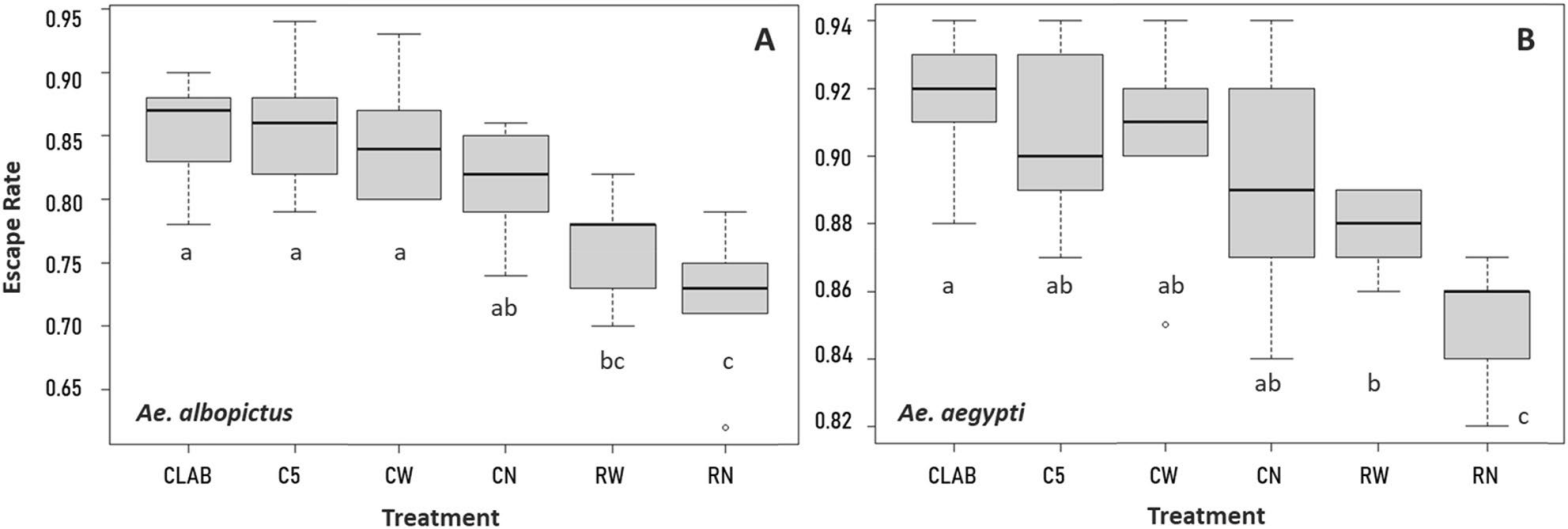
Escape rate of *Ae. albopictus* (**A**) and *Ae. aegypti* (**B**) adult males (3–4 days old) subjected to different treatments C_LAB_, C_5_, C_W_, C_N_, R_W_ and R_N_. C_LAB_ untreated adult males, C_5_ cold knockdown adult males, C_W_ cold knockdown, compacted and cooled adult males; C_N_ cold knockdown and compacted adult males; R_W_ cold knockdown, compacted, cooled and irradiated adult males; R_N_ cold knockdown, compacted and irradiated adult males. The boxplot shows the minimum and maximum values, median, and upper and lower quartiles. Different letters represent statistically significant differences of escape rates at *p* < 0.05 level among the different treatments.

The quality of the adult Aedes males after the different cold knockdown, compaction, cooling and irradiation treatments were measured using the IAEA flight test devise protocol^23^. The escape rate of the adult *Ae. albopictus* and *Ae. aegypti* males from the flight test device is shown in Fig. 3. The flight propensity of *Ae. albopictus* and *Ae. aegypti* adult males was significantly affected by the irradiation (− 0.531 and − 0.304; Table 2) and the compaction treatments (− 0.262 and − 0.246; Table 2) while cold-knockdown (immobilization) procedures did not affect the flight performance of the males of both species. The use of the cold ring during compaction and mass irradiation procedures significantly improved the escape rate of *Ae. albopictus* and *Ae. aegypti* adult males (0.255 and 0.160, Table 2).

**Table 2:** Fixed effects of the binomial generalized linear mixed model for the effect of knockdown, compaction, cooling and irradiation on *Aedes albopictus* and *Aedes aegypti* male flight ability (***p < 0.001, **p < 0.01, *p < 0.05).

Species	Fixed effects	Estimate	SE	z value	*p* ( >|z|)
*Ae. albopictus*	(intercept)	1.746	0.117	14.887	0.000 ***
	radiation	− 0.531	0.059	− 9.014	0.000 ***
	cooling	0.255	0.058	4.381	0.000 ***
	compaction	− 0.262	0.069	− 3.785	0.000 ***
*Ae. aegypti*	(intercept)	2.331	0.065	35.654	0.000 ***
	radiation	− 0.304	0.070	− 4.311	0.000 ***
	cooling	0.160	0.070	2.273	0.023 *
	compaction	− 0.246	0.084	− 2.939	0.003 **

#### Effect of treatments on male fertility

The residual fertility of adult Aedes males subjected to the different cold knockdown, compaction, cooling and irradiation treatments was analysed. As expected, the mean fertility rate (± SD) of *Ae. albopictus* adult males was only significantly affected by irradiation treatment (R_W_: 0.015 ± 0.003; R_N_: 0.015 ± 0.004; Table 3) with no effect observed from the other treatments (C_LAB_: 0.911 ± 0.075; C_5_: 0.915 ± 0.074; C_W_: 0.919 ± 0.062; C_N_: 0.914 ± 0.078; Table 3). Similarly to *Ae. albopictus*, the mean fertility rate (± SD) of *Ae. aegypti* males was affected by the irradiation treatments (R_W_: 0.007 ± 0.003; R_N_: 0.008 ± 0.002; Table 3) without any significant effect caused by compaction and cooling treatments (C_LAB_: 0.966 ± 0.017; C_5_: 0.961 ± 0.017; C_W_: 0.965 ± 0.013; C_N_: 0.957 ± 0.020; Table 3). The mean (± SD) radiation dose received by the adult *Ae. albopictus* and *Ae. aegypti* males was 54.93 (± 2.35) and 67.92 (± 2.99) Gy, respectively. The overall mean residual fertility rate of irradiated adult *Ae. albopictus* and *Ae. aegypti* males (1.46 ± 0.30% and 0.77 ± 0.23%, respectively) was significantly different (F_1,52_ = 3018, p < 0.001 and F_1,52_ = 55,855, p < 0.001) from the overall mean fertility rate observed in the conspecific unirradiated groups (91.49 ± 6.92% and 96.20 ± 1.70%).

**Table 3:** Fixed effects of the binomial generalized linear mixed model for the effect of cold knockdown, compaction, cooling and irradiation on Ae. albopictus and Ae. aegypti male fertility (“***” p < 0.001, “**” p < 0.01, “*” p < 0.05).

Specie	Fixed effects	Estimate	SE	z value	p ( >|z|)
***Ae. albopictus***	(intercept)	2.536	0.401	6.331	0.000 ***
	radiation	− 6.949	0.122	− 56.965	0.000 ***
	cooling	0.049	0.095	0.521	0.602
	compaction	0.029	0.087	0.337	0.736
***Ae. aegypti***	(intercept)	3.289	0.176	18.672	0.000 ***
	radiation	− 8.144	0.161	− 50.537	0.000 ***
	cooling	0.193	0.139	1.388	0.165
	compaction	− 0.156	0.134	− 1.162	0.245

## Discussion

The radiation treatment to sterilize insects is one of the most critical procedures for an effective large-scale AW-IVM programme that includes an SIT component. The sterilization process must be carried out at the most suitable stage of development of the insect, and must result in a homogeneous, stable and adequate level of sterility while minimizing the impact on the quality of the insects produced. Although irradiating males as adults has been shown to generate effective sterility levels with minimal biological quality costs^7,8,15,24–26^, irradiation of pupae has been preferred in the past due to the safer and easier manipulation of the pupae in water. However, irradiation of pupae *en masse* has recently shown inconsistent and unsatisfactory results^9^. Difficulties were also observed in different *Aedes* suppression trials for the standardization of adult male sterility and quality levels using pupal irradiation procedures at densities of 50,000–250,000 pupae per litre of water^11,27^. The radiation sterilization of mosquitoes as adults seems therefore desirable for an effective and sustainable implementation of mosquito operational SIT but requires the development of effective compaction and immobilization methods of large numbers of adult mosquitoes.

After the development and evaluation of effective procedures for compaction, cooling and irradiation of adult mosquitoes^21^, a 3D printed coolable canister was developed as part of a research project on developing new tools and procedures for mass-irradiation of adults. Results of this study showed that the phase change material (PCM) based cooling ring does not alter the dose distribution and homogeneously attenuated the absorbed dose resulting in an appropriate dose uniformity ratio (DUR = 1.1)^7^. The irradiation canister proposed can effectively hold and cold-immobilise about 100,000 adult mosquitoes at a compaction rate of about 120 males per cm^3^ for a duration of 30 min. The current radiation dose range for the reliable sterilization of these species as adults (50–70 Gy) can be delivered within this timeframe using most available irradiators with different dose rates.

The thermal shock treatment used in this trial to cold-anesthetize adult mosquitoes significantly reduced the survival of *Ae. albopictus* but not that of *Ae. aegypti*. As previously reported adult cold treatments affect the performance of both species with *Ae. albopictus* males more sensitive than *Ae. aegypti* at all cooling durations^17,28^. However, the effects of the chilling treatment are no longer detectable in *Ae. albopictus* male flight propensity 24 h after the cold knockdown treatment. The *Ae. albopictus* males tested when 3–4 days of age showed a lower flight ability than *Ae. aegypti* male of the same age when tested at 24h following the different treatments. However, the overall mean (± SD) flight performance of compacted, chilled and irradiated *Ae. albopictus* (R_W_: 76.8 ± 4.1%) and *Ae. aegypti* (R_W_: 87.7 ± 1.3%) males measured in our trials were similar or even higher than the flight performance previously reported after comparable treatments^23,29,30^, showing an excellent overall quality achieved with the use of the chilled irradiation canister.

Irradiation procedures can generally affect insect quality^31^, but the mass-rearing, transport and release procedures can likewise reduce the mating ability and field competitiveness, decreasing the efficiency of SIT programmes^32^. The radiation treatments of adult males during this trial reduced the survival and the flight ability in both species, but with a greater effect on *Ae. albopictus*. However, the cooling applied with the use of the cold ring during radiation can significantly reduce the physical damage suffered by the males of both species during mass irradiation procedures. As previously reported^15^, while irradiation, compaction and chilling carried out at the adult stage can impact flight ability and longevity, only irradiation significantly affected the sterility level in both species. The unavoidable variability observed in the absorbed dose from samples randomly placed in the different canister compartments during this trial allowed us to define 58 and 65 Gy as the effective doses (ED) to achieve a residual fertility (RF) of about 1% in *Ae. albopictus* (ED = − 6.74*RF + 64.77; R^2^ = 0.754; F_1,16_ = 49.1, *p* < 0.001) and *Ae. aegypti* (ED = − 11.13*RF + 76.51; R^2^ = 0.761; F_1,16_ = 50.8, *p* < 0.001)^5^ under mass irradiation conditions.

In addition to irradiation, male compaction significantly affected male quality in both species reducing longevity and flight performance. However, no significant differences were reported on these quality parameters between untreated (C_LAB_) and compacted and cooled males (C_W_) in both species. The use of the canister with the cold ring therefore strongly reduces the damage improving the adult male quality with significant increase in the survival rate, flight ability and likely their mating performance^29,33^. A marginal reduction of sterile male quality was observed with radiation sterilised *Ae. aegypti* adult males incubated for 24 h at similar compaction rates (80–100 males/cm^3^) and cooling temperature ranges (7–14 °C). The physical damage suffered by the adults in these trials was most likely due to mutual friction or improper long storage conditions rather than adult compaction^14,19^. In order to use the proposed canister for mass irradiation and subsequent transportation and release procedures it will therefore be important to avoid both excessive compaction and the presence of free space between cold immobilised males.

The use of this cooled canister has proven to be a useful tool during adult mass irradiation, especially when it is necessary to move the samples between different facilities or for irradiation with long exposure times. The cooling temperature inside the canister is critical and immobilization temperature below 6 °C has been previously shown to reduce the quality of adult *Aedes* males even with 1 h of exposure^18^. Temperatures below 6 °C were observed in the canister compartments in the first 20 min after chilling suggesting the need to let the cold ring warm up at room temperature before its use. After this time, the temperature (6.7–11.3 °C) and the relative humidity (68.9 – 73.9%) ranges observed in the canister over a period of 30 min after cold ring placement are appropriate for effective chilling and compaction procedures^21^. However, adult irradiation procedures carried out on compacted and cold anesthetized adult mosquitoes could be performed without the use of additional cooling devices for irradiation treatments shorter than 10 min. In the present study we confirmed that cold anaesthetised and compacted adult samples can remain chilled inside the proposed canister after the initial cooling treatment. However, this irradiation procedure is feasible only when the irradiation facility is in proximity of the mass rearing facility and the irradiator source has an adequate dose rate. Samples of approximately 1,000 *Ae. aegypti* adult males compacted inside plastic compartments of about 10 cm^3^ volume are routinely cold anesthetized and radiation sterilized without cooling devices at the IPCL using radiation times of less than 2 min. After being compacted, irradiated and shipped over long distances for 24 h at controlled temperatures (7–12 °C), adult *Ae. aegypti* males showed good survival and quality for their performance in SIT suppression trials^14,21^. However, samples of *Ae albopictus* adult males shipped over long distances under cooling conditions generally showed lower quality even for samples with less compaction rates^34^. Once again, immobilization and cooling conditions need to be carefully controlled, especially in *Ae. albopictus* to effectively manage adult handling procedures for sterilization, transport and release.

However, even if each mosquito species, handling procedures or irradiator type may require a dedicated study for the creation of an ideal model, the mass irradiation canister proposed and tested in this study represents a versatile initial solution for an immediate improvement in the quality and performance of adult sterile males to be used in larger scale SIT operational projects. Although this irradiation canister has shown excellent performance in maintaining and improving the quality of treated adults, an improvement in its thermal insulation could optimise the cooling period to avoid the exposure to variable or extreme temperatures. In addition, the use of a stable cold room with a working temperature above 5 °C could further improve the cold anaesthesia and insect handling procedures. Finally, the possibility of adjusting the cooling structures in the canister according to the radiation beam geometry would decrease the attenuation effects, maintaining the dose rate of the available irradiator. Future development of the canister will need to consider the possibility of irradiating the mosquitoes inside a mass rearing canister that will be subsequently used for transportation and loading of mechanised aerial release devises^20^.

It is also necessary to underline that the proposed canister can be easily replicated using plastic 3D printing technologies reducing prototyping and production costs. Access to 3D printers and to their products has seen a massive increase in recent years and the use of this technology could strongly expand the possibility to share models and develop advanced prototypes capable to strongly improve current mosquito SIT projects worldwide.

## Methods

### Mosquito colony and rearing procedures

The IAEA laboratory reference strains of *Aedes aegypti* (Juazeiro, Brazil) and *Ae. albopictus* (Rimini, Italy) strains were used in these experiments. These strains have been maintained for more than 10 years at the IPCL in Seibersdorf, Austria, using the IAEA guidelines for colony maintenance and mass-rearing of *Aedes* mosquito species^35^.

The rearing of these species was carried out under controlled climatic conditions for the larval (28 ± 2 °C; 80 ± 10 RH%; 14:10 L:D) and adult stages (26 ± 2 °C, 60 ± 10 RH%, 14:10 L:D) and rearing procedures were based on the IAEA guideline^36^ with a modified rearing schedule^23^. Pupae collected at day 7 after egg hatching were sexed using a mechanical, automated sorter (Guangzhou Wolbaki Biotech Co. Ltd., Guangzhou, China) with a female contamination rate of less than 1% in both species^37^. The male pupae selected were volumetrically aliquoted into 18 samples of about 1,000 males and allowed to emerge into large plastic laboratory cages (30 × 30 × 30 cm, BugDorm, DP1000, Taiwan) with constant access to a 10% sucrose solution. An equivalent number of samples with 50 virgin females were also selected under a stereomicroscope and placed into 18 small laboratory cages (17.5 × 17.5 × 17.5 cm, BugDorm, BD4M1515, Taiwan) with access to 10% sucrose solutions to estimate the fertility rate of the conspecific males coming from different treatments. All insects were maintained under adult rearing conditions as described above.

### Radiation sources and dosimetry

A gamma irradiator, Gammacell 220 (GC 220 Nordion Ltd., Kanata, Ontario, Canada) was used to test the irradiation canister and the effect of mass irradiation on the adult mosquitoes. Adult *Ae. albopictus* and *Ae. aegypti* mosquitoes aged 2 to 3 days were exposed to the target doses of 55 Gy^30^ and 70 Gy^38^ respectively. During the irradiation treatments, the irradiator had a dose rate of 48 to 46 Gy/min. Each irradiation time for the different doses was calculated based on the decay table of the isotopic source routinely corrected^7^. The dosimetry system used to verify the dose received by the samples was based on Gafchromic HD-V2 film (Ashland Advanced Materials, Bridgewater NJ, USA) following FAO/IAEA standard operating procedures^22,39,40^. Three 1 × 1 cm HD films were placed in paper envelopes (2 × 2 cm) and placed next to each sample during irradiation. The films were read with an optical density reader (DoseReader4, RadGen Ltd, Budapest, Hungary) after 24 h of development.

### Cooled irradiation canister

#### Description of the irradiation canister

**Figure 4:**
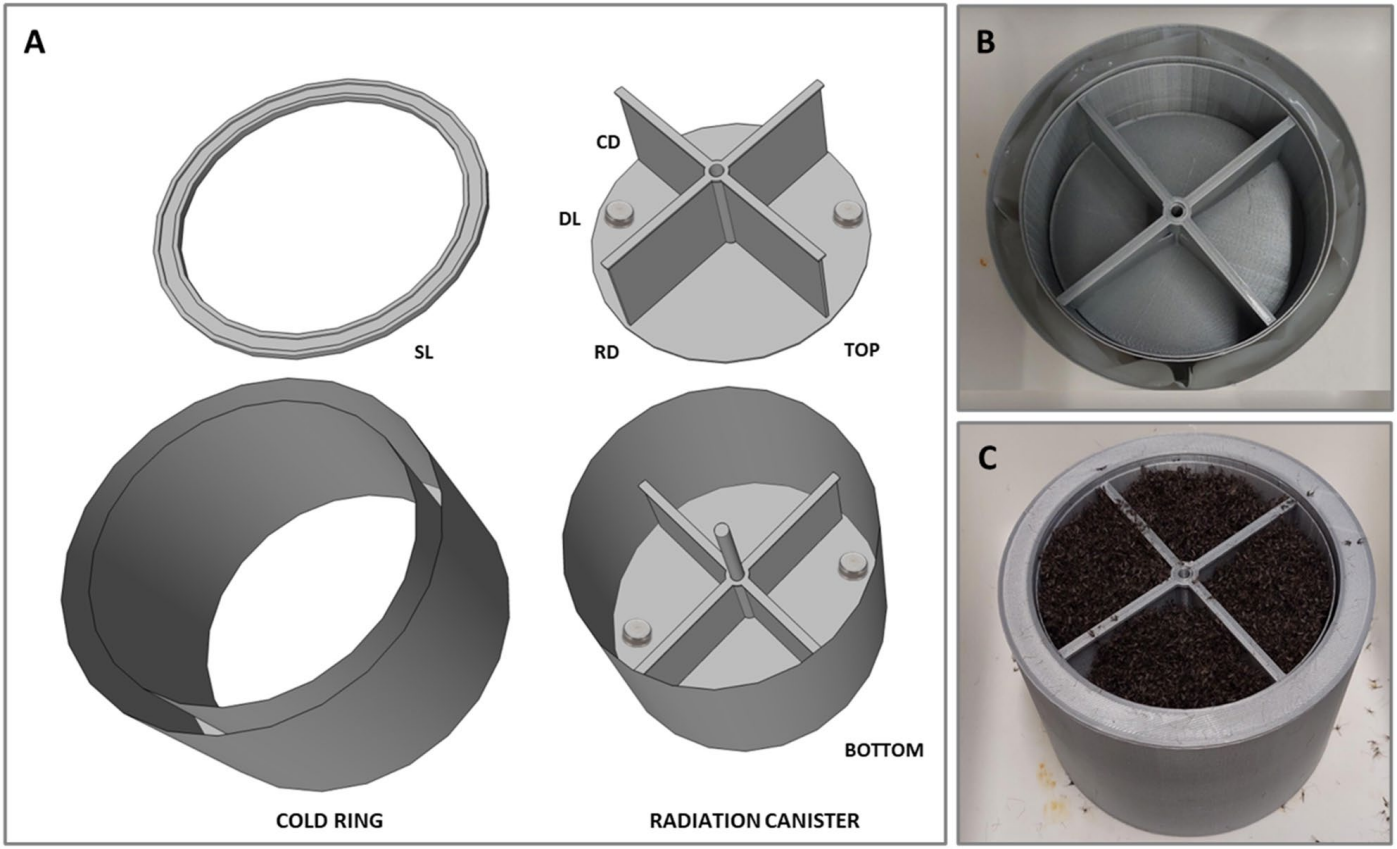
Technical drawing (**A**) showing the external cold ring with its snap lid (SL) and the internal irradiation canister with the upper (TOP) and lower compartments (BOTTOM) separated by a removable disk (RD). The position of the data loggers (DL) used to measure temperature and humidity in the top and bottom compartments is reported. The compartments have four sections each created by two removable cross dividers (CD); 3D printed plastic canister assembled with external cooled ring filled with phase change material packs (**B**); Irradiation canister fully assembled and filled with chilled adult mosquitoes (**C**).

A plastic canister made of polylactic acid (PLA) was created with a 3D printing technique (Ultimaker 3S, Ultimaker B.V., Geldermalsen, The Netherlands) and tested for mass irradiation of adult mosquitoes. The canister consists of two stacked compartments, each with four sections, that can be inserted inside a plastic ring (Fig. 4A). This ring (inner diameter = 12 cm, outer diameter = 14 cm) can be filled with cold-conditioned phase change material packs (PCM; ClimSel C7, Climator Sweden AB, Skövde, Sweden; Fig. 4B) to ensure a sustained, stable cooling effect throughout the entire canister. The canister tested (outer diameter = 12 cm, height = 9 cm), offers an overall usable volume of about 800 cm^3^ once the eight sections of the two stacked compartments are filled with an overall maximum load capacity of about 95,000–100,000 mosquitoes (compaction height of 4 cm; Fig. 4C). When used inside the irradiator, the aqueous polymer solutions of the PCM could be interposed between the radiation field, thereby attenuating the dose received by the samples. To mitigate the shielding effects of the cold ring during irradiation, the upper and lower parts of the canister are not in direct contact with the cooling surface, leaving these areas more exposed to external conditions. To improve the thermal isolation of the container, the canister was therefore integrated with a structure capable of elevating the canister bottom by 5 mm away from direct contact with the support surface. Several preliminary observations were conducted on the proposed prototype canister to assess its cooling capacity and the degree of attenuation of the radiation beam once used in a ^60^Co gamma irradiator, Gammacell 220.

#### Effect of the cold ring on temperature and relative humidity

The temperature and humidity in the container positioned inside the Gammacell irradiator (21 ± 0.5 °C, 60 ± 10 RH%) were recorded with and without the use of the cold ring. The cold ring was always maintained in the cold room (3.0 ± 1.0 °C, 70 ± 5.0 RH%) for at least 24 h before each observation. Environmental data were collected using four hydrochronic data loggers (DS1923-F5# iButton, Whitewater, WI, USA) with logging rate of one minute, positioned in two opposite sections of the upper and lower compartments of the canister (Fig. 4A, DL). Since adult mosquitoes do not emit significant metabolic heat when chilled and held in an immobile compacted state^17^, we opted to use dead mosquitoes to measure the environmental parameters in the irradiation canister. All measurements were carried out with the canister filled with dead mosquitoes stacked at a thickness of up to 4 cm in all compartment’s sections. The dead mosquitoes were kept in the cold room before being used in the various observations and regularly replaced to better simulate the moisture content of cold-anesthetized, live adult mosquitoes.

#### Effect of the cold ring on dose rate attenuation

Dose rates obtained inside the irradiation canister with and without the cold ring were measured using a Farmer type 0.18 cm^3^ free air ionization chamber (10X6-0.18, RadCal Corporation, Monrovia, CA. USA) in conjunction with a digitizer and electrometer (AccuDose Model 9660A) as a reference dosimetry system to measure the dose rate and accumulated dose at a designated reference position. The ion chamber system was calibrated by the John Perry Laboratory (St George’s University Hospital Trust, London) with traceability to the National Physical Laboratory with a calibration factor of 1.0 and uncertainty of 3.3% (k = 2) in the energy range 40–1250 keV. The absorbed dose measured inside each section and in the central point of the two compartments were registered with and without the cold ring. The dose time used to assess each measurement was equal to 1 min (dose rate 47 Gy/min). Because the sterility of *Aedes* adult males in air is not affected by adult density, which is close to air^30^, dose rate measurements were performed without filling the canister with dead mosquitoes to easily access each compartment’s sections.

### Knockdown, compaction, cooling and irradiation treatments

Between 2 to 3 days post-emergence, adult cages were deprived of the sugar solution sources and transferred to the cold room for a cold knockdown period of 10 min. After knockdown, mosquitoes were collected from the cages by gently shaking and dropping the mosquitoes inside a plastic tray covered with absorbent paper strips^21^. Males were then transferred into a dedicated mosquito plastic tube with both bottom and lid netted (inner diameter 1.4 cm, height 4 cm, volume 6 cm^3^) using a funnel in compacted batches of about 700–750 mosquito per tube (about 120 mosquito/cm^3^). Once a sample was obtained for each cage, excess mosquitoes were discarded and eliminated after compaction. Three compacted samples were then placed within the irradiation canister in different compartments and sections. All compartments of the canister were filled with dead mosquitoes to a depth of 4 cm to simulate the filling and compaction of the entire canister. The container including the three tube samples was subsequently transferred alternately inside the Gammacell 220 irradiator chamber under the following conditions:

i) with the cold ring and maintained inside the irradiator for 30 min (C_W_ treatment),
ii) without the cold ring and maintained inside the irradiator for 30 min (C_N_ treatment),
iii) with the cold ring, irradiated and maintained inside the irradiator for 30 min (R_W_ treatment),
iv) without the cold ring, irradiated and maintained inside the irradiator for 30 min (R_N_ treatment).

When irradiated, the samples were accompanied with dosimetric films and the irradiation treatment was carried out approximately halfway through the 30-min observation period. This observation period was selected to simulate irradiation time with lower dose-rate irradiators. After the different treatments the canister was moved back to the cold room where each of the three samples were separated from the dead and divided into two subsamples and placed in large laboratory cages respectively (about 450–500 adult males) for survival analysis and in small laboratory cages (about 250–300 adult males) for the evaluation of male residual fertility and flight capacity.

Two additional treatments were also carried out using mosquitoes that were subjected to only the cold knockdown (immobilisation) treatment (C_5_ treatment) and mosquitoes that were kept after emergence in the adult rearing room (C_LAB_ treatment). For these two additional treatments, the subsamples necessary to evaluate survival, fertility and flight capacity were obtained by collecting mosquitoes from the initial emergence cages using a mouth aspirator (Model 612, John W. Hock Company, Gainesville, FL, US). Three biological replicates, each with three technical repetitions were performed for each treatment and species tested.

#### Effect of treatments on male survival

Adult samples from the different treatments were kept in large laboratory cages with continuous access to 10% sucrose solution and observed for 28 days to evaluate male longevity. Dead males were recorded 3 times per week and the number of male mosquitoes alive on day 28 were recorded before discarding the cages.

#### Effect of treatments on male flight ability

Adult samples from the different treatments were kept in small laboratory cages with continuous access to 10% sucrose solution for 24 h before being tested for their flight ability using the IAEA flight test device and protocol^23,29^. Batches of 100 adults of 3 to 4 days of age were transferred into the flight test device for 2 h. Adult escaped and trapped inside the flight test device were then counted to define the male flight ability at 24 h after each treatment conditions. Flight ability was calculated by dividing the number of adults escaped by the total number entered in the flight tube^23^.

#### Effect of treatments on male residual fertility

Additional batches of 50 males per treatment were selected from each small laboratory cage at 24 h from treatment and placed inside separate small laboratory cages containing 50 conspecific virgin females isolated after pupal sexing procedures. At four days after introducing the males, a blood meal of citrated (0.14% w/v sodium citrate E331, Almi GmbH, Oftering, AT) pork blood was offered to the females. Oviposition cups containing water and filter papers were added to each cage two days after blood feeding and eggs were collected three to four days after the cups were introduced. Egg papers were left to dry slowly and stored at high humidity for at least one week before hatching. The eggs collected were counted under a stereomicroscope and hatched overnight in bacterial broth solution. After submersion, egg papers were checked under the stereomicroscope to count the hatched and unhatched eggs^40^.

### Statistical analysis

Statistical analyses were carried out with R version 4.1.0^41^ using RStudio^42^ and with IBM SPSS Statistic v.23 (IBM Corporation, Armonk, NY) with a significance level (α) of 5%.

General linear model (GLM) univariate factorial analysis followed by post hoc Tukey test were used to analyse the influence of compartment position (top or bottom) and cooling conditions (with or without cold ring) on the temperature and relative humidity parameters measured inside the treatment canister. ANOVA models were applied to compare the mean absorbed dose rate inside the upper and lower compartments of the canister with and without the use of the cold ring. The best model was selected based on the lowest corrected Akaike information criterion (AICc). Normality and homoscedasticity were confirmed with Shapiro and Levene tests respectively.

The mixed-effects Cox model (“coxme” function in “survival” package) fit by maximum likelihood with mosquito time to death as response variable, treatments (knockdown, compaction, chilling and irradiation treatments each with two levels: present or absent) and their interaction as fixed effects and repetition as a random effect, was used to analyse the survival of each mosquito species. Survival graphs were built using the ggplot2 package. Kaplan–Meier survival analysis was also carried out for all treatments groups and data sets were compared using the Mantel-Cox log-rank test.

The male flight ability, as a direct marker for insect quality, was analysed using a generalized binomial linear mixed-effects model (GLMM) fit by maximum likelihood (Laplace approximation) with a logit link, with the escape rate (proportion of flyers) as dependent variable, treatments and their interaction as fixed effects and the replicates nested with technical repetition as a random effect for each species tested.

A binomial GLMM fit by maximum likelihood was also used to analyse the dose response curves of *Ae. aegypti* and *Ae. albopictus* adults, considering the egg hatch rates as response variable, treatments and their interaction as fixed effects and the replicates as a random effect for each species tested.

For validation, the full models were checked for overdispersion (using Bolker’s function; package bblme)^43,44^ and for normality and homogeneity of variances on the residuals^45^. The stepwise removal of terms followed by likelihood ratio tests (LRTs) or based on the lowest value of Akaike’s Information Criterion (AICc) was used for model simplification. The minimal adequate model retained only factors that significantly reduced explanatory power (p < 0.05) when removed^46^.

### Supplementary Information

Supplementary Information.

## Data Availability

All data generated or analyzed during this study are included in this published article. Raw data are provided in the supplementary information.
